# C-reactive protein as a biomarker of severe H1N1 influenza

**DOI:** 10.1007/s00011-018-1188-x

**Published:** 2018-10-04

**Authors:** Denitsa Vasileva, Alaa Badawi

**Affiliations:** 1 Faculty of Arts and Science, University of Toronto, Toronto, ON Canada; 2 Public Health Risk Sciences Division, Public Health Agency of Canada, 180 Queen Street West, Toronto, ON M5V 3L7 Canada; 3 Department of Nutritional Sciences, Faculty of Medicine, University of Toronto, FitzGerald Building, 150 College Street, Toronto, ON M5S 3E2 Canada

**Keywords:** Systematic review, H1N1, C-reactive protein, Inflammation, Influenza

## Abstract

**Background:**

C-reactive protein (CRP) is an acute-phase reactant downstream of the pro-inflammatory cytokines released during influenza infection. However, the role of this inflammatory marker in influenza severity and complications is yet to be elucidated.

**Objectives:**

We aim to systematically review and evaluate the levels of CRP in severe and non-severe H1N1 influenza cases and assess its utility as a biomarker in predicting the severity of infection.

**Methods:**

We conducted a comprehensive search in Ovid MEDLINE, Ovid MEDLINE (R) Epub ahead of Print, Embase and Embase Classic to identify human studies reporting measurements of CRP levels in patients infected with H1N1 influenza at various levels of disease severity.

**Results:**

Our search identified ten studies eligible for inclusion in this systematic review. The results of the data analysis show that the average CRP levels upon diagnosis were significantly higher (*P* < 0.05) in patients who developed severe H1N1 influenza compared to their counterparts with a no severe disease. Furthermore, levels of CRP were associated with the degree of H1N1 severity. Subjects with H1N1-related pneumonia and patients who were hospitalized or died of the disease complications, respectively, had 1.4- and 2.5-fold significantly higher CRP levels (*P* < 0.05) than those with no severe disease outcome.

**Conclusion:**

CRP levels have been consistently shown to be significantly higher in H1N1 influenza patients who develop a severe disease outcome. The resuts of the present study suggest that serum CRP can be employed—in combination with other biomarkers—to predict the complications of H1N1 influenza.

**Electronic supplementary material:**

The online version of this article (10.1007/s00011-018-1188-x) contains supplementary material, which is available to authorized users.

## Introduction

Influenza has been a major source of morbidity and mortality worldwide ever since its discovery in the sixteenth century [1]. Influenza viruses encompass a large and highly diverse group of viruses belonging to the *Orthomyxoviridae* family [2] that usually contain a single-stranded, negative-sense enveloped RNA genome [3]. In 2009, the H1N1 strain of the influenza virus—commonly known as swine flu—was responsible for global influenza pandemic affecting more than 170 counties and over 19,000 laboratory-confirmed deaths [4]. The true death toll of the pandemic was, however, estimated to be anywhere from 150,000 to > 500,000 deaths [4, 5].

The H1N1 virus invades the body either via skin contact with a contaminated surface, membrane contact with aerosols containing the virus or inhalation of airborne viral particles [6]. Upon entry, the virus binds to epithelial cells in the respiratory tract to interfere with the host protein synthesis and induce virion replication followed by host cell death [7]. Most patients infected with H1N1 influenza display mild clinical manifestations including cough, fever, headache and malaise; symptoms that are usually resolved within 7–10 days due to the host’s immune response to infection [7]. A small proportion of infected patients, however, develop more severe complications including pulmonary and cardiac infections such as pneumonia and myocarditis with subsequent hospitalization and/or death [7].

Upon infection of the epithelial cells with the H1N1 virus, various innate immunity-related factors are recruited to mediate the synthesis and secretion of human type I and type III interferons (IFNs) and a range of pro-inflammatory cytokines such as interleukins (IL)-6, IL-1β and tumor necrosis factor (TNF)-α [8]. The significant increase in the synthesis of these cytokines (so called cytokine storm) was observed in H5N1 influenza and was linked to severe tissue and organ damage in the infected subjects and to the likelihood of developing an array of disease complications [9]. C-reactive protein (CRP) is a downstream acute phase reactant protein that complements the innate immune response [10, 11]. It is produced as a result of the increased synthesis of pro-inflammatory cytokines to activate the complementary immune response [11]. Therefore, serum CRP levels has been often used as a laboratory marker of inflammation [10]. Significant increase in the levels of CRP were reported in patients with H1N1 and in severe cases of infection [12]. Despite many findings suggesting a role of CRP in severe outcome of influenza infection, no systematic analysis was conducted to examine the association between the two conditions and the potential of CRP to be employed as a biomarker to predict the severity of infection. Therefore, the objective of the present study was to systematically review and evaluate the utility of CRP as a biomarker to predict the severe outcome of H1N1 infection.

## Methods

### Literature search

A systematic review was undertaken in compliance with the PRISMA (Preferred Reporting Items for Systematic Reviews and Meta-analysis) framework (Supplementary Table 1) [13]. The literature search was conducted in Ovid MEDLINE, Ovid MEDLINE (R) Epub ahead of Print, Embase and Embase Classic using the search terms (MeSH): “CRP” or “C-reactive protein” AND “Influenza” (Supplementary Table 2). The time period of the articles in the publication search was from the inception of the databases to end of September 2017. Only English language articles focusing on human subjects were included. Review papers, letters to the editor, vaccine studies, editorials, case reports, animal studies, conference abstracts and duplicated studies were excluded. Studies were only included if they focused on H1N1 influenza in adults and reported quantitative levels of CRP in H1N1 patients. Reference lists of included studies were manually checked for relevant records for inclusion.

### Inter-reviewer agreement

Two reviewers (DV and EC) independently reviewed the abstracts yielded from the search to determine those eligible for full-article review and inclusion in the study. Disagreements regarding study inclusion were resolved by an arbitrator (AB). Percentage agreement and Cohen’s Kappa (*κ*) score were calculated [14] and interpreted as outlined by Landis and Koch’s benchmarks for κ statistic [15]: poor (< 0), slight (0.00–0.20), fair (0.21–0.40), moderate (0.41–0.60), substantial (0.61–0.80), and excellent (0.8–1.0). The percentage agreement between the two reviewers was 91% with a substantial *κ* of 0.72 (95% CI: 0.64–0.80).

### Data extraction and analysis

Data extracted from the selected studies included author’s name, year of publication, country of study, dates of patient recruitment, cohort sample size (subsequently divided into male and female), the average age estimates (based on mean, median or mid-point of range) of study cohorts, frequency of clinical symptoms (%), and the levels of CRP (mg/L). These information were extracted from each of the selected studies for the entire study subjects (overall) and  for non-severe and severe H1N1 cases. Severe H1N1 cases was classified as any course of H1N1 influenza that deviates from the ordinary disease progression—i.e., self-contained symptoms that are resolved within 14 days. In the “severe” influenza cases, we included patients who developed viral or bacterial pneumonia, underwent hospitalization, admitted to intensive care unit (ICU), suffered from respiratory or cardiovascular complications and/or died. Non-severe H1N1 was classified as H1N1 influenza cases that followed the normal self-limiting disease course. The cohort within each study was categorized into severe and non-severe groups based on the above definitions. For each subgroup, the weighted average age and CRP level (upon diagnosis) were calculated. In addition, the weighted average CRP level was calculated for each type of complication and compared to the levels in patient with non-severe H1N1 infection. Statistically significant differences were calculated at *p* < 0.05 using *t* test for comparison.

## Results

### Search results

**Fig. 1:**
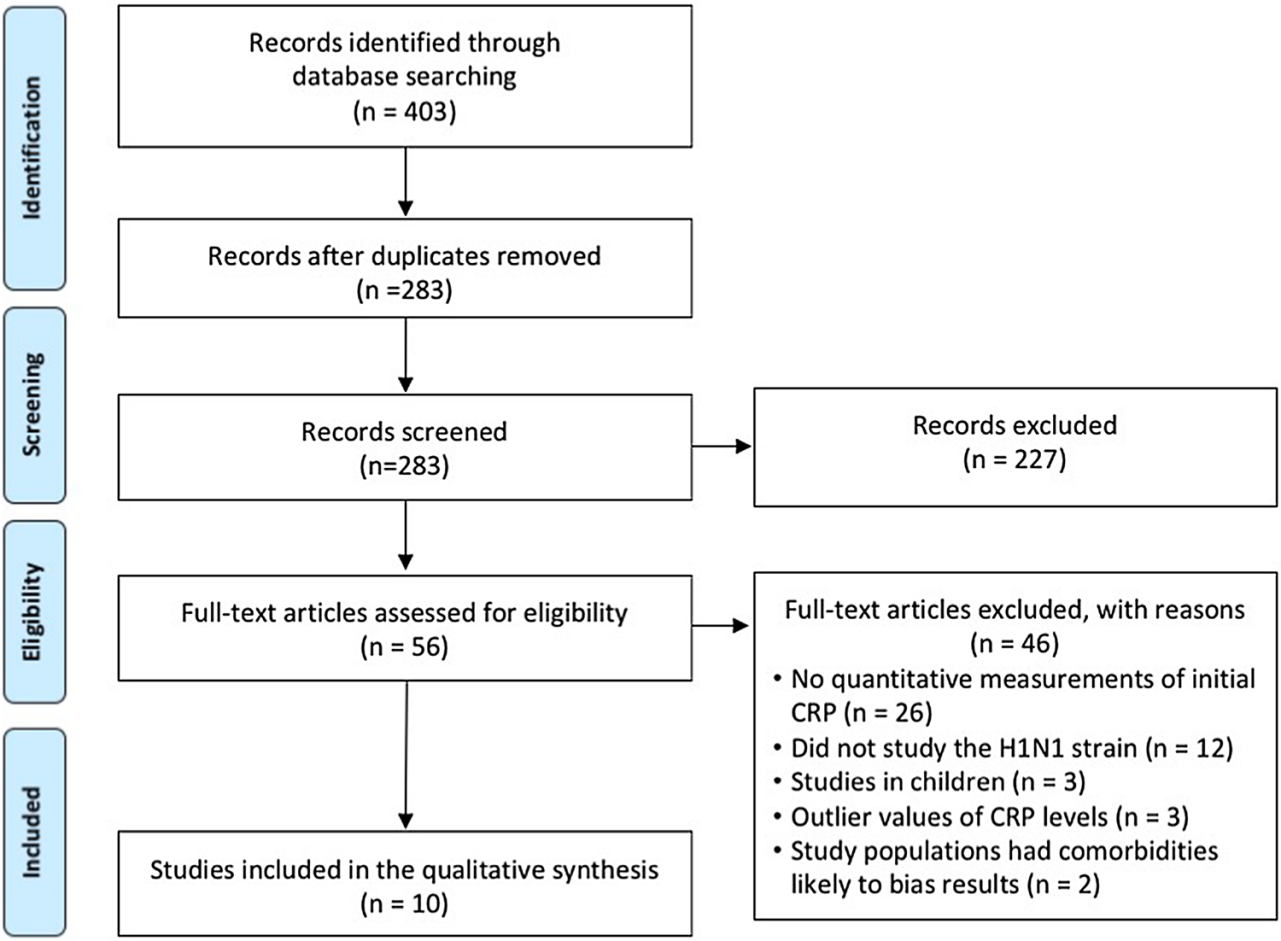
Flowchart of study selection and systematic literature review process. The flow diagram describes the systematic review of literature evaluating the levels of CRP as potential biomarkers of severe H1N1 cases in human. Full texts of 56 studies were examined and 10 unique reports were identified to be included into the qualitative assessment and analysis

The process used to conduct the present systematic review is shown in Fig. 1. The initial database search yielded 403 records (supplementary Table 3) which met the search inclusion/exclusion criteria (Supplementary Table 2). Following the removal of duplicate studies, 273 records remained and were screened by abstract review. Of these, 56 abstracts were eligible for full-text review. Based on the inclusion/exclusion criteria, 46 full-text articles were excluded (Fig. 1). Briefly, 26 articles did not include quantitative measurements of CRP levels, 12 focused on types of influenza other than H1N1, 3 examined children, 2 evaluated CRP in study populations with significant comorbidities that could bias the CRP levels and 3 reports with CRP levels that were significantly far above the clinically and physiologically recognized range (outliers). Ten studies examining the initial (i.e., upon infection) levels of CRP in patients with H1N1 were, therefore, selected for inclusion in the present review [12, 16–24].

### Population characteristics

The total number of subjects in the selected studies was 1307 patients (Table 1) with a sex ratio (male: female) of 0.51 and a weighted overall average age (± SD) of 41 ± 10 years (range 31–63 years). Total number of participants in the 10 selected studies ranged from 59 to 293 cases. The small number of studies reported here is the result of our stringent search strategy and narrow inclusion/exclusion criteria. Our sole focus was mainly on the studies that explicitly state levels of CRP in H1N1-infected patients at diagnosis. The majority of the cases were Asians and Europeans and mainly recruited between mid 2009 to early 2010, i.e., at the peak of the 2009 H1N1 flu pandemic. Based on our definition of H1N1 severity (see above), we identified a total of 485 cases (sex ratio: 0.49, average age: 44 ± 17) and 822 cases (sex ratio: 0.44, average age: 54 ± 7) of H1N1 patients to, respectively, have severe and non-severe infection outcomes. Fever (70%, 95% CI 68–73%), myalgia (37%, 95% CI 33–37%) and headache (15%, 95% CI 14–15%) were the most prevalent clinical symptoms in the examined cases. Five of the selected studies did not report the clinical manifestations of H1N1 influenza in the examined patient [18, 20, 22–24].

**Table 1:** Characteristics of the selected studies

Study ID	Country	Recruitment dates (mm–yy)	Number of subjects									Age (years)			Frequency of clinical symptoms (%)			Comments
			Overall			Severe			Non-severe			Overall	Severe	Non-severe	Fever	Myalgia	Headache	
			Total	M	F	Total	M	F	Total	M	F							
Mulrennan et al. [16]	Australia	07.09–08.09	70	35	35	35	20	15	35	15	20	38	38		49	26	9	**Design:** retrospective **Purpose:** the utility of CURB-65 severity index in predicting severe pneumonia in H1N1-infected patients
Zimmerman et al. [17]	Israel	2009	191	96	95	17			174			43			14			**Design:** observational **Purpose:** the utility of CRP levels in predicting the severity of H1N1 infection
Milosevic et al. [18]	Serbia	05.09–03.10	63	26	37	46	18	28	17	8	9	35	36	32				**Design:** observational **Purpose:** the utility of non-specific inflammatory markers (e.g., CRP) in predicting the severe outcome of H1N1 infection
Canak et al. [19]	Serbia	10.09–02.10	293	152	141	13			280			33	28		100	41	16	**Design:** retrospective **Purpose:** describe the clinical and laboratory features of H1N1 patients who required ICU admission
Kok et al. [20]	Australia, New Zealand	2009	145	72	73	145	72	73				47						**Design:** retrospective **Purpose:** the risk of severe H1N1 in obese patients
Sohn et al. [21]	South Korea	06. 09–12.09	59	23	36	59	23	36				31	31		90	36	15	**Design:** prospective cohort **Purpose:** clinical characteristics of H1N1-related pneumonia compared to community acquired pneumonia
Wi et al. [22]	South Korea	06.09–11.09	104	52	52	24	10	14	80	42	38	51	62	55				**Design:** retrospective **Purpose:** the correlation CRP and the respiratory support needs of H1N1 patients
Hong et al. [12]	South Korea	10.11–05.12	123	52	71	40	22	18	83	30	53	63	74	57	90			**Design:** prospective case–control **Purpose:** to characterize the clinical and laboratory characteristics in hospitalized H1N1 patients
Feng et al. [23]	China	06.09–01.10	173	110	63	90			83			32						**Design:** retrospective (registry) **Purpose:** to determine the correlation between lung HRCT and levels of CRP in H1N1 patients
Morton et al. [24]	UK	11.10–01.11	86			16			70									**Design:** retrospective **Purpose:** factors that indicate safe discharge of H1N1 patients
Total/Weighted average ± SD			1307	618	603	485	165	174	822	95	120	41 ± 10	44 ± 17	54 ± 7	70 ± 36	37 ± 6	15 ± 3	

### Levels of CRP in severe and non-severe cases of H1N1 influenza

**Fig. 2:**
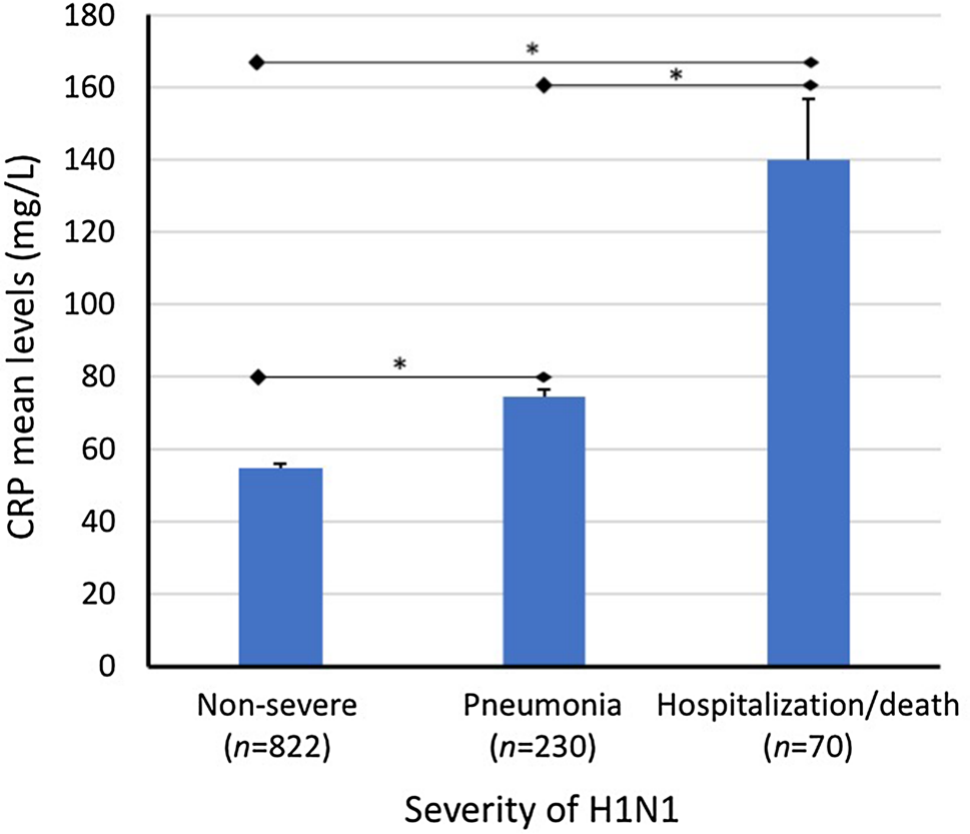
Levels of CRP in relation to the degree of H1N1 severity. Values represent the average initial levels of CRP (± SE) in non-sever [12, 16–19, 22–24] cases of H1N1 and in cases with viral and bacterial pneumonia as secondary complication of the infection [16, 18, 21, 23] and in hospitalized (and ICU) and H1N1 patients who died [17, 19, 22, 24]. Significant differences (*) at *p* < 0.05, Student’s *t* test, are shown between the different levels of disease severity

Levels CRP in H1N1 patients varied by threefold across the studies (range 34–111 mg/L) as shown in Table 2. The overall average level of CRP in H1N1 patients was 68 ± 28 mg/L. When patients were stratified based on the severity of H1N1 infection, those with severe disease outcome had about 1.6-fold higher average levels of CRP compared to their counterparts with the non-severe infection (90 ± 44 vs. 55 ± 31 mg/L, *p* < 0.005). Among the 230 patients who developed viral or bacterial pneumonia as severe complications, the average level of CRP (± SD) was 75 ± 31 mg/L (Fig. 2). Patients who developed a severe H1N1 complications and required hospitalization and admission to ICU and those who died (*n* = 70) had 140 ± 138 mg/L average levels of initial CRP. The differences between the levels of CRP in non-severe, H1N1-pneumonia and H1N1-hospitalization/death subgroups were statistically significant (*p* < 0.05). Host-related factors such as age and gender did not show any significant correlation with the levels of CRP in either the entire examined cases or within the non-severe or severe subgroups (data not shown).

**Table 2:** Average levels of CRP in severe and non-severe H1N1 cases

Study ID	Mean CRP levels (mg/L)			Outcome
	Overall	Severe	Non-severe	
Mulrennan et al. [16]	72	97	51	The CURB-65 severity index score did not predict severe influenza, but CRP levels were higher in cases with severe outcome
Zimmerman et al. [17]	47	123	40	Initial CRP levels were useful in predicting the need for ICU admission and/or mechanical ventilation in H1N1-infected patients
Milosevic et al. [18]	49	142	32	CRP was significantly higher in patients who developed pneumonia
Canak et al. [19]	98	198	93	Elevated CRP level was the only factor that significantly differed between patients admitted to ICU and those who did not
Kok et al. [20]	111	111		CRP is not an adequate marker of pneumonitis in obese H1N1 patients
Sohn et al. [21]	52	52		Patients with H1N1-related pneumonia had lower CRP levels compared to those with community acquired pneumonia
Wi et al. [22]	88	139	72	Patients needing respiratory support had higher levels of CRP than those who did not (but not as an independent predictor)
Hong et al. [12]	40	89	17	CRP level could be a useful marker for prediction of complications in H1N1 patients
Feng et al. [23]	34	46	13	As the lung lesions (visible on the HRCT) increased in size and number, the levels of CRP were elevated
Morton et al. [24]	44	112	27	Low CRP level was a significant indicator of safe discharge
Weighted average ± SD	68 ± 28	90 ± 44	55 ± 31	*p* < 0.005 between severe and non-severe H1N1 cases (Student’s *t* test)

## Discussion

The present study was conducted to systematically evaluate the levels of CRP in patients with H1N1 influenza at different degrees of severity. The majority of the studies examining the initial levels of CRP in H1N1 influenza suggest that they significantly increase in patients who develop severe complications of influenza compared to those with no severe disease outcome [12, 16–19, 22–24]. Indeed, infection with H1N1 influenza elicits a complex response from the innate immune system leading to a rapid increase in the synthesis of the pro-inflammatory cytokines [8]. These are small signaling protein that mediate their effects at both the primary site of infection (primary cytokines) and in the downstream immune response (secondary cytokines) [8]. Primary cytokines, such as interferons (IFNs), act to limit and contain viral replication and are involved in the viral clearance during H1N1 infection. IL-1β is another cytokine shown to improve the outcome of H1N1 infection by stimulating the CD8T cells and their activity while IL-6 was found to be essential for preventing death of neutrophil cells caused by the viral infection [8]. Despite their protective effect, a dysregulated over production of these cytokines was implicated in the pathophysiology of severe infection [25]. Multiple studies have suggested that overproduction of cytokines (cytokine storm) and the overexuberant immune response can lead to a wide range of severe complications [8, 25, 26]. In animal and cell culture models, among other cytokines, over-synthesis of interleukins (such as IL-1β and IL-6) and TNF-α were shown to play a major role in the development of a wide range of severe disease outcomes [reviewed in 8, 11]. Furthermore, cytokine storm has been implicated previously both in severe H5N1 and H7N9 influenza [26, 27] where excessive immune activation, particularly the complement activation mediated by CRP, was reported [27].

CRP is an acute phase reactant activated by cytokines to trigger the complement component of the immune system [10, 11, 27]. Previous studies in influenza A have shown that overactivation of the complement cascade contributes to the cytokine storm and can play a role in the pathogenesis of, rather than protection against, influenza [26, 27]. As CRP acts an intermediate factor in the relationship between over-synthesis of serum cytokines and severe influenza, it can be suggested that it can be employed as a biomarker to predict the likelihood of an H1N1-infected patient to develop severe H1N1 influenza. The results of the present study support this assumption as the CRP levels were significantly elevated in patients with the severe disease compared to their counterparts of the non-severe H1N1. Furthermore, CRP appears to account for the disease severity regardless of age or gender. Factors such as age and gender in the present study were not correlated with CRP irrespective to severity (data not shown). Additionally, CRP levels showed a strong correlation with the degree of severity (Fig. 2). Taken together, these observations may implicate serum CRP levels as a factor contributing to the severe H1N1 infection and underline its possible utility in predicting the disease complications.

Although the present study is the first systematic review for the association between CRP and severity of influenza, it has several limitations. The studies included in this report have a wide between-study variance (fivefold) in their sample size. This difference is even wider between studies stratifying the cases into those with non-severe (16-fold) and severe (tenfold) infection. Moreover, there is a large variability in the average CRP levels between the selected studies that varies by 3-, 5- and 7-fold, respectively, in the overall, severe and non-severe cases. The large between-studies difference in the sample size and CRP levels render comparison challenging among the different disease conditions and/or examined populations. Similarly, investigating a particular association between the levels of CRP and a given clinical conditions can be hindered by the large between-studies differences. Further, the small number of studies and their limited geographic region may levy limitations in terms of the ability to generalize the utility of CRP as a marker for severe H1N1 influenza. Moreover, the selected studies provided only limited information on comorbidities, genetic and environmental factors that might be confounding factors that influence the CRP levels. This particular limitation highlights the need to develop larger prospective studies to evaluate the role of CRP in the severity of influenza and its utility in predicting disease outcome while considering a wide range of host-related modifiable (e.g., environmental) and non-modifiable (e.g., genetic) confounders. Furthermore, as this study focuses on H1N1 influenza, the association between CRP levels and severity of infection may not be extended to other type of influenza strains and infections.

In conclusion, the present study provides evidence that CRP levels upon diagnosis is significantly higher (on average) in patients who develop severe complications of influenza compared to those who present a non-severe course of the disease. Although this study cannot establish a causal link between higher levels of CRP and severe H1N1 influenza, it may suggest monitoring H1N1 patients with high CRP level upon diagnosis. At present, there are few clear strategies to determine which H1N1 influenza patients are likely to develop severe outcome. Given the contradicting evidence for the value of some genetic markers, e.g., polymorphism at the *IL-1β* locus, in predicting severity [28, 29], a commonly tested laboratory marker such as CRP may prove useful in rapidly identifying patients predisposed to severe influenza when combined with other predictive biomarkers. Early risk prediction of severe disease consequence is an effective approach to advance healthcare systems as it has the potential to provide knowledge-based evidence for differential case management that can improve patient outcome.

## Electronic supplementary material

Below is the link to the electronic supplementary material.

Supplementary material 1 (PDF 248 KB)
