# Supplementary material for: C-reactive protein as a biomarker of severe H1N1 influenza
Source: Inflamm Res. 2018 Oct 4;68(1):39–46. doi: 10.1007/s00011-018-1188-x (PMC6314979; doi:10.1007/s00011-018-1188-x)
Supplement: Supplementary file 1 — Supplementary material 1 (PDF 248 KB) [file 11_2018_1188_MOESM1_ESM.pdf]

Supplementary Table 1. PRISMA Checklist [13].

Section/topic	#	Checklist item	page #
		**TITLE**	
Title	1	Identify the report as a systematic review, meta-analysis, or both.	1
		**ABSTRACT**	
Structured summary	2	Provide a structured summary including, as applicable: background; objectives; data sources; study eligibility criteria, participants, and interventions; study appraisal and synthesis methods; results; limitations; conclusions and implications of key findings; systematic review registration number.	2
		**INTRODUCTION**	
Rationale	3	Describe the rationale for the review in the context of what is already known.	4-5
Objectives	4	Provide an explicit statement of questions being addressed with reference to participants, interventions, comparisons, outcomes, and study design (PICOS).	Sup Table 2
		**METHODS**	
Protocol and registration	5	Indicate if a review protocol exists, if and where it can be accessed (e.g., Web address), and, if available, provide registration information including registration number.	No
Eligibility criteria	6	Specify study characteristics (e.g., PICOS, length of follow-up) and report characteristics (e.g., years considered, language, publication status) used as criteria for eligibility, giving rationale.	6
Information sources	7	Describe all information sources (e.g., databases with dates of coverage, contact with study authors to identify additional studies) in the search and date last searched.	6
Search	8	Present full electronic search strategy for at least one database, including any limits used, such that it could be repeated.	Sup Table 3
Study selection	9	State the process for selecting studies (i.e., screening, eligibility, included in systematic review, and, if applicable, included in the meta-analysis).	Sup Table 3
Data collection process	10	Describe method of data extraction from reports (e.g. piloted forms, independently, in duplicate) and any processes for obtaining and confirming data from investigators.	6-7
Data items	11	List and define all variables for which data were sought (e.g. PICOS, funding sources) and any assumptions and simplifications made.	7
Risk of bias in individual studies	12	Describe methods used for assessing risk of bias of individual studies (including specification of whether this was done at the study or outcome level), and how this information is to be used in data synthesis.	7
Summary measures	13	State the principal summary measures (e.g. risk ratio, difference in means).	7
Synthesis of results	14	Describe the methods of handling data and combining results of studies, if done, including measures of consistency (e.g. I^2) for each meta-analysis.	None
Risk of bias across studies	15	Specify any assessment of risk of bias that may affect the cumulative evidence (e.g. publication bias, selective reporting within studies).	None
Additional analyses	16	Describe methods of additional analyses (e.g. sensitivity or subgroup analysis, meta-regression), if done, indicating which were pre-specified	None
		**RESULTS**	
Study selection	17	Give numbers of studies screened, assess for eligibility, and included in the review, with reasons for exclusion at each stage, ideally with a flow diagram.	Figure 1
Study characteristics	18	For each study, present characteristics for which data were extracted (e.g. study size, PICOS, follow-up period) and provide the citations.	Tables 1-2
Risk of bias within studies	19	Present data on risk of bias of each study and, if available, any outcome level assessment (see item 12).	None
Results of individual studies	20	For all outcomes considered (benefits or harms), present, for each study: (a) simple summary data for each intervention group (b) effect estimates and confidence intervals, ideally with forest plot.	None
Synthesis of results	21	Present results of each meta-analysis done, including confidence intervals and measures of consistency.	None
Risk of bias across studies	22	Present results of any assessment of risk of bias across studies (see Item 15)	None
Additional analysis	23	Give results of additional analyses, if done (e.g. sensitivity or subgroup analyses, meta-regression [see item 16]).	None
		**DISCUSSION**	
Summary of evidence	24	Summarize the main findings including the strength of evidence for each main outcome; consider their relevance to key groups (e.g., healthcare providers, users, and policy makers).	11
Limitations	25	Discuss limitations at study and outcome level (e.g., risk of bias), and at review-level (e.g., incomplete retrieval of identified research, reporting bias).	11
Conclusions	26	Provide a general interpretation of the results in the context of other evidence, and implications for future research.	11-12
		**FUNDING**	
Funding	27	Describe sources of funding for the systematic review and other support (e.g., supply of data); role of funders for the systematic review.	14

Supplementary Table 2: PICOST Table.

Component	Criteria
Population	Patients who were infected with H1N1 influenza up to September 2017
Intervention	No intervention criteria
Comparison	- Compare initial CRP levels in patients with severe H1N1 influenza to those with non-severe H1N1 influenza
Outcome	- Changes in the course of H1N1 influenza depending on initial levels of CRO
Study Design	- English-language studies exclusively focused on human subjects.<br>- Observational studies (including retrospective chart review).<br>- Cohort (>5 patients) studies.<br>- Cross-sectional studies.
Time	Up to September, 2017
Selection Criteria for Full Screening	Inclusions:<br>- Human studies of patients with H1N1 infection which specify CRP values for these patients<br>Exclusions:<br>- Review articles, letters to the editor, case-reports, editorials, conference abstracts.<br>- Duplicate study.<br>- Non-English.<br>- Animal studies; studies not conducted on humans.<br>- Vaccination trials.<br>- Studies without abstracts.<br>- Family-based studies.

Supplementary Table 3. Search Strategy.

Category	#	Searches	Results
Details: Ovid MEDLINE: Epub Ahead of Print, In-Process & Other Non-Indexed Citations, Ovid MEDLINE® Daily and Ovid MEDLINE® <1946-Present>, Embase Classic+Embase <1947 to 2017 September 08>, AMED (Allied and Complementary Medicine) <1985 to September 2017>			
Search terms	1	exp influenza/	244164
	2	exp CRP/	113593
Combination	3	1 and 2	554
Limitation	4	limit 3 to human	476
Limitation	5	limit 4 to humans	476
Limitation	6	limit 5 to English language	403
De-duplication	7	remove duplicates from 6	293
De-duplication	8	remove duplicates from 7	283
